# A review: The nutrition components, active substances and flavonoid accumulation of Tartary buckwheat sprouts and innovative physical technology for seeds germinating

**DOI:** 10.3389/fnut.2023.1168361

**Published:** 2023-07-05

**Authors:** Yulu Dong, Nan Wang, Shunmin Wang, Junzhen Wang, Wenping Peng

**Affiliations:** ^1^College of Biological and Food Engineering, Anhui Polytechnic University, Wuhu, China; ^2^Academy of Agricultural Science Liang Shan, Liangshan, China

**Keywords:** Tartary buckwheat, sprouts, nutrition components, flavonoid, physical technology

## Abstract

Compared with the common grain, Tartary buckwheat enjoys higher nutritional value. Some distinctive nutrition associated with physiological activity of Tartary buckwheat is valuable in medicine. In addition, it’s a good feed crop. In the paper, the main components (starch, protein, amino acid, fatty acid and mineral) and polyphenol bioactive components in Tartary buckwheat and its sprouts were reviewed, and the accumulation of flavonoids in sprouts during germination, especially the methods, synthetic pathways and mechanisms of flavonoid accumulation was summarized. The research on bioactive components and health benefits of Tartary buckwheat also were reviewed. Besides, the applications of innovative physical technology including microwave, magnetic, electromagnetic, ultrasonic, and light were also mentioned and highlighted, which could promote the enrichment of some active substances during seeds germination and growth of Tartary buckwheat sprouts. It would give a good support and benefit for the research and processing of Tartary buckwheat and its sprouts in next day.

## 1. Introduction

Grain is one of the world’s most momentous cash crops, with an annual yield approximately 2.5 × 10^12^ kg (1). Sprouted grains and legumes are considered to be more nutritious and digestible than unsprouted varieties. Some studies even suggest that, compared with non-germinated cereals seed, the proportions of active components, like protein, γ-aminobutyric acid (GABA), free amino acid, flavonoid, and phenol, are significantly increased in germinated cereals, while the contents of unsaturated fatty acid (UFA), carbohydrate, and mineral of seedlings are reduced. Germinated cereals were closely related to abundant nutritional value and health functions (2). Sprouted grains, which can be fresh-cooking or taken advantage as a high value health ingredient after superfine grinding powder (3), are becoming increasingly popular with consumers in food, medicine, health products, cosmetics and other fields.

*Fagopyrum tataricum* (L.) Gaertn. (Tartary buckwheat), belonging to the family *Polygonaceae* and genus *Fagopyrum*, is widely consumed around the world (4). Tartary buckwheat is an annual herb dicotyledonous plant with erect, branched, green or purplish, pinstriped stems. Leaves have long stem, leaf blade is broadly triangular, seeds are conical ovate, sharp upper part, rounded lower part, pericarp rough, dark brown, with three deep furrows (5). Tartary buckwheat is one of the minor cereals in Asia, Europe, North America and South Africa. As an important economic grain crop in China, it has been cultivated for more than 2000 years. Tartary buckwheat is a kind of medicinal and edible plant resource with high nutritional and medicinal value. Furthermore, it’s a typical representative of the homologous culture of medicine and food.

Tartary buckwheat is abundant in a variety of nutrients and bioactive substances, such as protein, flavonoid, unsaturated fatty acid, cellulose, mineral and vitamin and others (6, 7). Compared with other grains, Tartary buckwheat possesses variety of nutritional ingredients and more balanced nutrients (8), the details of Table 1 (9, 10) and Table 2 (9–17) are as follows. Being known as the 21st century nutritional food, it’s generally 76.7% carbohydrate (including 5.8% dietary fiber), 12.2% protein and 4% fat, etc. In the recent centuries, Tartary buckwheat is mainly consumed in the form of flour, bread, vinegar, tea and sprouts in Japan, Italy and China, and whole Tartary buckwheat seeds play great effect (18). Recent studies have shown Tartary buckwheat sprouts is rich in flavonoids and other polyphenols, so which displays some delightful of biological activities, such as anti-oxidation (19–21), anti-cancer (22, 23), anti-inflammation (24), lowering blood pressure (25), reducing blood sugar (26), lowering blood fat (25) and improving immunity (6, 27). It is used as a dual-purpose crop and good functional food raw material, so research and development of related products with Tartary buckwheat sprouts has attracted more and more people (28).

**Table 1:** Comparison of nutritional components between Tartary buckwheat and common grains.

Composition	Tartary buckwheat	Brown rice	Millet	Corn (Grits)	Black rice	Wheat	Quinoa	Oat	Rice
Fat (g)	2.70	3.84	3.75	2.33	3.03	3.09	7.26	9.96	1.08
Protein (g)	9.70	11.45	12.44	8.59	9.66	11.13	15.69	14.81	7.58
Carbohydrate (g)	60.20	71.39	72.25	72.98	76.33	75.08	54.07	50.32	—
Calcium (mg)	39.00	12.76	9.30	20.06	17.54	17.21	37.90	—	13.45
Iron (mg)	4.40	0.66	1.19	0.26	2.47	1.82	2.23	2.34	2.34
Zinc (mg)	2.02	0.95	1.35	—	1.32	1.12	1.67	1.62	1.68
Selenium (ug)	5.57	1.34	2.68	1.01	1.09	8.08	2.13	3.46	2.12
Vitamin B1 (mg)	0.28	0.35	0.34	0.03	0.34	0.41	0.38	0.87	0.12
Vitamin E (mg)	1.73	0.78	3.56	0.38	0.23	1.82	2.34	1.01	0.46
Vitamin B2 (mg)	0. 16	0.12	0.11	0.02	0.15	0.17	0.32	0.22	0.05

This is the nutrient content of 100 grams of edible food.

**Table 2:** Comparison of fatty acid and functionally active components of Tartary buckwheat and common grains.

Fatty acid	Tartary buckwheat	Brown rice	Millet	Corn (Grits)	Black rice	Wheat	Quinoa	Oat	Rice
Palmitic (C16:0)	13.7	18.41	6.95	22.19	18.81	21.53	9.75	16.56	20.8
Stearic (C18:0)	1.6	0.45	6.17	—	0.54	0.50	—	—	2.11
Oleic (C18:1)	35.2	46.01	14.75	19.87	38.14	15.99	26.70	46.86	32.72
Linoleic (C18:2)	39.6	32.64	68.50	57.94	40.40	55.39	49.95	35.05	39.47
Linolenic (C18:3)	5.7	2.1	3.03	—	1.90	4.48	9.19	1.54	1.35
Arachidonic (C20:0)	1.2	—	—	—	—	—	1.68	—	—
Saturated	25.3	19.17	13.63	22.19	19.57	22.28	10.48	16.56	26.46
Unsaturated	74.5	80.83	86.37	77.81	80.43	77.72	89.52	83.44	73.54
Unsaturated/saturated	2.94	4.22	6.37	3.51	4.11	3.49	8.54	5.04	2.78
Total flavonoid	125.33	93.97	23.82	1.48	0.05	0.05	0.9	0.07	0.286
Total polyphenol	107.24	96.29	82.22	1.27	0.52	0.08	1.44	0.13	1.25

The units are % in the table.

Germination is a popular method among health lovers in recent years. It’s also a common practice for improving the digestibility and nutritional value of seeds, grains, nuts, or legumes (29). Affected by many elements, including seed vigor, temperature and time, as well as some external chemical or physical stimuli such as hormones, visible light, ultraviolet light, ultrasound (US), microwave (MW), and magnetic field (MF), electromagnetic field (EMF) (30), and static electric field (SEF), seed germination is a complex series of physiological and biochemical processes. Controlling germination can effectively enhance food quality in nutritional value and flavor (31). Besides, promoting the germination of grain seeds, non-thermal technologies of US, MW and MF produce a series of specific effects and physiological and biochemical changes. Compared with hormones and exogenous additives and/or heat treatment, non-heat treatment is not only limited to altering the physical and chemical properties of grains, but can also upgrade nutritional and functional ingredients and reduce anti-nutritional factors by inducing associated biochemical transformation (32). Therefore, in recent years, a great deal of new non-thermal physical treatment technologies, containing ultra-high voltage, MW, MF, high-voltage pulse electric field, ionizing radiation and pulse have been gradually applied to the field of germination, and have achieved fruitful research results (33).

This paper provided an overview of the changes of the main components (starch, protein, polyphenol, amino acid, fatty acid and mineral) in Tartary buckwheat and its sprouting seedlings. It focused on the synthesis and enrichment ways of flavonoids, the main active components in Tartary buckwheat, as well as germination processing methods. This paper also reviewed research activities related to the germination of Tartary buckwheat both domestically and abroad. In order to provide reference for full exploitation and utilization of Tartary buckwheat, we collected previous research and summarized this review.

## 2. The main nutritional components of the Tartary buckwheat sprouting

Nowadays, more and more people pay attention to their health, for it is one of the most important things we should do to get more natural health benefits from food. Being rich in vitamins and nutrients, whole grain foods such as oat, buckwheat, Tartary buckwheat, barley, wild rice and quinoa have been proven that they can reduce the risk of heart disease, type 2 diabetes, obesity and some cancers. Furthermore, they make great effects in improving gut health and other diseases.

The excellent nutritional qualities and remarkable functional activities of Tartary buckwheat have attracted close attention of scholars (Table 3). At present, many studies have shown that the structure, properties, *in vitro* digestibility and functional activities of Tartary buckwheat, which can be improved by heat, humidity, light and cold, etc. As the same time, the flavonoids ingredients can be enriched by increasing the gene expression of key enzymes, thus opening up a new way for the development of new functions of Tartary buckwheat and its applications in food industry (58).

**Table 3:** Physiological function of Tartary buckwheat sprout.

Sprout or its components	Physiological function	Researchers
Whole grains	Decreased risk of type 2 diabetes	Qiu et al. (26)
Sprouts	Decreased the glycemic index	Bhinder et al. (34)
	Lowered blood glucose	Fonteles et al. (35)
	Improved oxidative damage in HePG2 cells	Jeong et al. (36)
	Increased the activities of enzymes SOD and POD	Bian et al. (37)
	Improved antioxidant capacity and anti-inflammatory activities through inhibiting cyclooxygenase-2 and lipoxygenase activities	Almuhayawi et al. (38)
	Higher inhibitory activity by the production of proinflammatory mediators such as nitric oxide and cytokines including tumor necrosis factor-α, interleukin- (IL-) 6, and IL-12	Nam et al. (24)
	Produced more free DCI	Hu et al. (39)
	Increased the DCI content (up to 9-fold)	Qin et al. (40)
	Increase α-glycosidase activity to released more free DCI	Wang et al. (41)
Hull	Anti-cancer (Extraction with 70% ethanol)	Kim et al. (22) and Ren et al. (23)
Starch	Decreased blood glucose, increased the insulin sensitivity, improved the metabolic disorder of glucose and lipid in type 2 diabetic patient	Qin et al. (42)
	RS decreased blood glucose, increased the insulin sensitivity	Molska et al. (43)
	Decreased the glycemic index	Qin et al. (42)
	Reduced serum lipid levelsa and promoted the proliferation of Lactobacillus (Bifidobacterium, Lactobacillus, and Enterococcus)	Liu et al. (44)
Protein	Anti-oxidation and cholesterol-lowering	Zhou et al. (45)
	Inhibited OḤ (about 17.55 u/MG) and the capability of scavenging IC_50_ by 1.372 mg/mL	He et al. (46) and Luo et al. (47)
	Decreased plasma total cholesterol by 45%	Zhang et al. (48)
	Reduced serum lipid levelsa and promoted the proliferation of Lactobacillus (Bifidobacterium, Lactobacillus, and Enterococcus)	Liu et al. (44)
Phenolics	Anti-inflammatory by reducing GPDH activity and NO production	Lee et al. (49)
	Anti- cancer	Kim et al. (22) and Ren et al. (23)
	Anti-oxidation, lowering blood pressure	Kreft (25)
	Antioxidant capacity (Quercetin scavenging activity of DPPH and superoxide anion radical is about 148 and 183% of vitamin C)	Li et al. (50)
	Anti-diabetic effect	Bao et al. (51)
	Protected hypertension, ameliorates insulin-induced vasodilatation and insulin signaling pathways	Hou et al. (52)
	Antioxidant capacity, inhibition of a-glycosidase and a-amylase, and starch digestibility	Peng et al. (53)
Amino acid	Anti-aging and improvement of metabolism	Tomotake (54)
Fatty acids	Prevention of cardiovascular disease, antibacterial and anti-inflammatory	Calder (55), Hassinen et al. (56), and Hogan et al. (57)

### 2.1. Tartary buckwheat starch

The content of Tartary buckwheat starch (TBS) is the highest proportion in all of the carbohydrates, and its structure and properties also are complex. Current research on TBS focus on structural modification and bioactivity studies (59). Some researchers revealed the compositions, structure and properties of TBS, which indicated the amylose content of Tartary buckwheat was approximately 20% ~ 28%, and the resistant starch (RS) content was approximately 13.06% ~ 23.07% (59–61). While the amylopectin had super-long unit chain (DP > 100), the long chain starch content of amylopectin was 12% ~ 13%, and the crystalline type was A-type polymorphism with the size range of 2 ~ 15 μm. By changing the molecular structure of TBS, it can improve its properties or endow it with new functions (62). At present, there are many reports about the modification of TBS, such as pressure heat method, damp heat method, enzyme method, high pressure treatment and so on. The results showed that the solubility, swelling power, crystal morphology and functional properties of TBS modified by alcohol-alkali method, drying method and ball milling method were improved to some extent (63).

The properties and *in vitro* digestibility of TBS using heat-moisture treatment (HMT) and annealing (ANN) was revealed, under different treatments, the amylose content, relative crystallinity, water absorption and gelatinization temperature increased (64, 65), but the solubility and swelling power of TBS decreased with temperature (66), the total hydrolysis and RDS (rapidly digestion starch, 20 min after digestion) decreased, while SDS (slow digestion starch, 20 ~ 120 min after digestion) and RS increased (67). Both ANN and HMT can effectively improve the properties and *in vitro* digestibility of TBS. In addition, the thermal stability, functionality and application value of TBS are increased significantly.

The impacts of ultra-high pressure (HHP) treatment on Tartary buckwheat starch’s properties were displayed in two similar studies. HHP treatment could cause TBS particles to sag and adhere, and could gradually lose its original shape, but its crystalline type was still typical A-type. Other study reported that HHP treatment could induce the surface roughness of TBS particles, the amylose content and gelatinization temperature increased significantly with the increase of pressure (68), while the relative crystallinity, swelling force, hardness and viscosity decreased. However, compared with natural starch, HHP modified TBS hydrolysis degree was lower, RDS content was lower, SDS and RS content were higher. The structure, physical and chemical properties, microbial properties and *in vitro* digestibility of TBS were changed by HHP treatment (69).

Some studies found that TBS was not easily digested by amylase because of its close association with flavonoid. Therefore, TBS mainly exists in the form of RS, and contains more SDS and less RDS. RS is not digested in the intestine but it is fermented by gut microbes to produce short-chain fatty acids (SCFA) when RS enters the large intestine (70). Part of SCFA is absorbed by the intestine and enters into the blood circulation system, which is utilized by the body through a series of biochemical reactions, thus exerting certain physiological functions (71). It has also been proved that RS can decrease the postprandial blood glucose, increase the insulin sensitivity and improve the metabolic disorder of glucose and lipid in type 2 diabetic patients (42). It was reported that TBS could decreased the plasma total cholesterol (TC) and total triglycerides (TG) by 22.86 and 63.16%, respectively, when the mice were fed with a high-fat diet (44). Through the above, it is concluded that TBS has advantages in lowering cholesterol, preventing diabetes and cardiovascular diseases and controlling cancer.

The changes of properties of Tartary buckwheat starch during germination were studied. The results implied that the starch loss increased (4.4% ~ 5.9%) moderately and scanning electron microscope showed that the porosity around starch granules improved in the process of germination. Germination process had no obvious influence on the X-ray diffraction pattern, while the crystallinity percentage of starch increased with germination rate increasing. Besides, the process of germination upgraded the solubility of buckwheat starch, but had no obvious effect on swelling power. Therefore, a number of functional properties of Tartary buckwheat starch were improved with the treatment of moderate germination (72). A study showed water absorption and foaming ability of the obtained sprout flour increased, but foaming stability decreased after 48 h of Tartary buckwheat germination. The compound nutrient flour G′ and G″ increased with the prolonged germinated (96 h) Tartary buckwheat flour and the addition of germinated Tartary buckwheat flour into rice flour. The glycemic index of muffins with germinated (24 h ~ 48 h) Tartary buckwheat flour was lower than that of non-germinated Tartary buckwheat flour, but the glycemic index of Tartary buckwheat increased when germinating for 96 h (34).

### 2.2. Tartary buckwheat protein

Being an important nutrient of Tartary buckwheat, Tartary buckwheat protein (TBP) is rich in multiple amino acids and the proportion of amino acids is balanced (73). The results showed that the contents of low molecular weight protein and high molecular weight protein in TBP were relatively low, and more than three quarters (76.8%) of the proteins were mainly distributed between 10 ~ 60 kDa (74). TBP is mainly composed of glutenin, globulin, albumin and gliadin (61), the proportion of four proteins in total protein of Tartary buckwheat is 33.73, 14.08, 42.65 and 9.54%, respectively (75). It was found that the protein content decreased with the duration of seeds germination time. Accompanied by the protein content reduction, amino acids in Tartary buckwheat seedlings accumulated. It implied that the accumulation of amino acids was the result of protein degradation, thus improving the digestibility of buckwheat protein (45). The previous report confirmed that MW pretreatment at varying power and time had modulating influence on the structure, functional characteristics, and antioxidant capacity of germinated TBP. Compared with native germinated TBP, MW pretreatment promoted the structure expansion of germinated TBP, improved the content of free sulfhydryl groups in germinated TBP, the intensity of the ultraviolet absorption peak (*p* < 0.05) and random coil contents, and decreased the proportion of α-helix and β-folding in secondary structure. Furthermore, some properties of germinated TBP including solubility (24.37%), water-holding capacity (38.95%), emulsifying activity index (17.21%), emulsifying stability index (11.22%), foaming capacity (71.43%), foaming stability (33.60%), the *in vitro* protein digestibility (5.56%) and antioxidant capacities were significantly improved (*p* < 0.05). The functional properties of germinated TBP were optimized by MW treatment of 300 W/50 s (76).

Studies revealed that TBP had low digestibility and physiological functions such as anti-oxidation and cholesterol-lowering (45), and the capability of inhibit hydroxyl radical of TBP was 17.55 u/MG and the capability of scavenging IC_50_ was 1.372 mg/mL, which indicated that TBP had good *in vitro* antioxidant capacity (46, 77). The effect of TBP on plasma TC in hypercholesterolemic hamsters was surveyed, the result showed TBP could increase the excretion of total bile acid by 2.63 times and decrease the plasma TC by 45%, which indicated that TBP was an active component to decrease the total cholesterol activity of plasma (48). TBP could increase the activities of total Superoxide dismutase (SOD) (about 5.8% ~ 23.64%) and Glutathione peroxidase (GSH-Px) (about 15.3% ~ 65.04%) and decrease the level of liver malondialdehyde (MDA) (about 38.78% ~ 1.22%). Besides, it could lower blood sugar and protect the liver with histopathology studies. The outcomes have shown TBP enjoys a large of protective effects on hyperglycemia and liver injury in mice fed a high-fructose diet (78). Besides, it was reported that TBP could decreased TC and TG by 1.788, 52.05%, respectively, while inhibit the growth of E.coli in the gut of mice and promote the proliferation of Lactobacillus when the mice were fed with a high-fat diet (44).

### 2.3. Tartary buckwheat amino acids

Tartary buckwheat is abundant in high-quality protein, which contains 19 types of amino acids in proper proportions. So it has important research prospects in health functions, such as anti-aging and improving metabolism (54). Results revealed that all essential amino acids (EAAs) essential were present in Tartary buckwheat flours. In all buckwheat varieties, the contents of AAs differed significantly (*p* < 0.005). Among these EAAs, buckwheat varieties were characterized by high contents of leucine, followed by phenylalanine, lysine, and threonine (ranged from 743.66 to 1278.87, 398.48 to 877.96, 300.06 to 736.93 and 298.76 to 879.80 mg/100 g, respectively) (79). Germination can promote the variety of amino acids in Tartary buckwheat seedlings to become more diverse and rich in contents. The total amino acids contents of Tartary buckwheat decreased by 13.7% (notable Met+Cys and Phe + Try) after 72 h of germination (80), which were closely related to the transformation of nutrients in the early stage of seed germination. Tyrosine and L-Phenylalanine were involved in the early reaction of phenylpropane metabolism in plants (81), and their changes were closely related to the accumulation of plant secondary metabolites. During plant growth, proline metabolism was closely related to the production of reactive oxygen species (Ros). Ros is considered to be an important messenger in response to stress. Therefore, the increase of proline contents is generally considered to be an anti-stress response of plants in response to stress (82).

The study found, when after 48–72 h of germination of Tartary buckwheat, the content of EAAs of lysine, leucine and phenylalanine and GABA in sprout flour increased 2.3% ~ 24.77, 14.18% ~ 19.19, 10.66% ~ 21.45 and 27.48% ~ 40.89%, respectively (34). The effect of MW regulation on the variety and quantity of amino acid synthesis during the growth of buckwheat seeds was investigated. The results showed that under 300 W/50 s MW treatment, with the extension of the germination time of Tartary buckwheat seeds, the germination decreased first and then increased. Compared with sprouts without MW pretreatment, the cystine content of 5-day sprouts increased the most (158.62%), followed by tyrosine (38.71%), alanine (29.62%), proline (27.27%), valine (25.68%), histidine (24.32%), lysine (24.18%), threonine (24.14%), and phenylalanine (23.73%). EAAs and total amino acids (TAAs) content increased by 25.92 and 24.65%, respectively. In addition, compared with the other MW treatment groups, the benefits of EAAs and TAAs were the most significant at 300 W/50 s, and the EAAs/TAAs value was the highest under this condition (35.04%) (83).

### 2.4. Tartary buckwheat phenols

Tartary buckwheat is rich in flavonoids, phenolic acids, quinones and other polyphenols. Flavonoid is one of the most abundant and important bioactive substances in Tartary buckwheat, mainly including rutin, quercetin and procyanidin (84). Tartary buckwheat flavonoids (such as rutin, myricetin and quercetin) have various biological activities and pharmacological effects, it is not only beneficial to cardiovascular and cerebrovascular health, but also has anti-inflammatory, antibacterial, antioxidant and antiviral properties (22, 23). Tartary buckwheat has great research value and future application in the food and pharmaceutical industries for its high contents in flavonoid. Rutin is a unique and abundant flavonoid in Tartary buckwheat. And it was found that the rutin content of Tartary buckwheat seeds was 30–150 times higher than that of common buckwheat seeds (85). Anthocyanin, a special kind of flavonoids with high antioxidant capacity, can accumulate in Tartary buckwheat and provide red pigment in Tartary buckwheat sprouts. In addition, it offers important nutrients and resists abiotic stresses to help Tartary buckwheat adapt to adverse environments (86).

It has confirmed that Tartary buckwheat flavonoid has the antioxidant and antidiabetic properties, in which rutin and quercetin are important active substances (87). Tartary buckwheat flavonoids had different scavenging abilities on various types of free radicals, and all three flavonoids (rutin, quercetin, isoquercetin) had strong activities of radical scavenging for DPPH (2,2-Diphenyl-1-picrylhydrazyl), but which had weak scavenging ability to hydroxyl and superoxide anion radicals. Quercetin had the highest biological activity. Its scavenging activity of DPPH and superoxide anion radical was about 148 and 183% of vitamin C, respectively, (50). The same results of ABTS (2,2′-Azino-bis (3-ethylbenzothiazoline-6-sulfonic acid) diammonium salt), DPPH and FRAP (ferric reducing antioxidant potential) *in vitro* also showed that Tartary buckwheat flavonoid had strong antioxidant capacity. Further studies have shown that increasing the consumption of glucose and the accumulation of glycogen content significantly in HepG2 cells, Tartary buckwheat flavonoid has distinct anti-diabetic influence (87). Hou et al. (52) investigated the effects of Tartary buckwheat flavonoid on vascular insulin sensitivity in spontaneously hypertensive rats, and the results showed TBF (Tartary buckwheat flavonoids fraction) could protect hypertension by attenuating vascular insulin resistance and oxidative stress. It indicated the chronic TBF diet may ameliorate insulin-induced vasodilatation and insulin signaling pathways in the mesenteric arterioles of spontaneously hypertensive rats, illustrating that TBF could be used for the treatment of hypertension.

Some research reported (88) Tartary buckwheat flavonoid could increase the activity of GSH-Px and SOD in mice livers, decrease the level of non-esterified fatty acid (NEFA) and MDA in mice livers and the activity of alanine aminotransferase (ALT) and aspartate aminotransferase (AST) in serum. Therefore, it can prevent vascular dysfunction and liver injury caused by high trimethylamine-N-oxide. The experiments confirmed that Tartary buckwheat flavonoid could prevent inflammation caused by obesity (49). Other study showed the flavonoid had effects on antioxidant capacity, inhibition of *a*-glycosidase and *a*-amylase, and starch digestibility (53). Besides, it was reported that the feed of Tartary buckwheat flavonoid could decreased content of TC and TG by 0.60 and 0.91 mmol/l, respectively, when the mice were fed with a high-fat diet (44). Hence, it has a good role in some filed for scavenging free radicals, anti-oxidation and anti-aging.

The dehulled seeds underwent germination, the total phenolic content (TPC) and antioxidant capacity of DPPH in sprout was 29.5 mg GAE/g dry weight (DW) and 29.87 TE/g DW, respectively, which was increased by 42.23 and 32.58% compared to hulled seeds. In addition to orientin and vitexin, high levels of other phenolic compounds (such as isoorientin and rutin by 1,402 and 967 μg/g DW, respectively) were detected in peeled germinated seeds. The data showed that buckwheat seeds, which were peeled and germinated, as a dietary source of phenolic compounds, had great application potential in functional foods and were beneficial to health (2).

During germination of Tartary buckwheat, the seeds leapt from a hypopus state to a dynamic state of frequent metabolic activity, during which respiration increased, the variety, quantity and activity of enzymes also increased significantly. It kept the physiological metabolism of seeds at a high level and accelerated the biological transformation based on enzymatic reaction (89). Germination effectively increases the digestibility and bioavailability of protein and starch, inhibits contents of toxic, harmful or anti-nutrient elements (90), and raises contents of some bioactive substances such as GABA (up to 143.20 mg/100 g) and rutin (up to 739.9 mg/100 g) (91), free amino acids, phenolics and flavonoid (45). Particularly, the germination has more notably effects on rutin contents and makes it reached to a peak value (12055.78 μg/g DW) (92), which can significantly improve the nutritional value and biological activity of Tartary buckwheat. Therefore, the germination treatment of Tartary buckwheat is of great significance, and the application of effective techniques to promote the germination of Tartary buckwheat is particularly important.

A lot of studies have shown that the content and activity of flavonoid in the germinated buckwheat can increase with different treatments. Tartary buckwheat can effectively promote the accumulation of flavonoid by light treatment. A report confirmed that all three treatments of UV-A (365 nm) UV-C (254 nm) after exposure to blue light and their combination treatments had influenced on flavonoid contents (rutin, quercetin) and total flavonoids in Tartary buckwheat, the rutin content under the combined irradiation of blue light and UV-C (BL + UV-C) was 13.5% higher than that of (UV-C + BL) (93). Sprouts irradiated 12 h with UV-C, UV-A and blue light after cultivated in darkness for 60 h, the total flavonoids content in sprouts reached approximately to 47, 46, 28 mg/g DW, respectively, which was 56.1, 51.2, 24.2% higher than that in darkness, respectively. Some physical methods of processing of US, MF and MW can also improve the concentration of flavonoids. The previous study demonstrated that ultrasound power, treatment time and other conditions could significantly affect the content of flavonoids, at the 6th day after incubation, the total flavonoids content in the seedlings treated with US for 30 min was up to 9.16 g/100 g, which increased by 0.64 times (94). Another study showed that the total flavonoids content in Tartary buckwheat sprouts was 9.46 g/100 g, increasing by 69.71% under the treatment of ultrasonic power 280 W, temperature 30°C and time 30 min (95). All of the MF and MW irradiation had significant alterations on the flavonoids content, the MF stimulation could induce the increase of total flavonoids content. The total flavonoids content reached the maximum value of 62.90 mg/g and increased by 43.21% when germinated for 5 days under 0.3 T magnetic field intensity (96). Similar to above study report, it was proved that 600 W MW irradiation could significantly increase the total flavonoids content in Tartary buckwheat sprouts (97). Bhinder et al. (34) confirmed that the antioxidant capacity of flour increased after 72 h of germination as a result of the accumulation of free flavonoids and phenolic acids. Although free rutin was the main polyphenol and reached to 88.1 mg/kg, the increment after germination was higher in kaempferol (2.42 mg/kg), catechin (39.59 mg/kg), vitexin (6.68 mg/kg) and p-coumaric acid (7.67 mg/kg).

### 2.5. Tartary buckwheat fatty acids

Tartary buckwheat is rich in multiple essential fatty acids like linoleic acid and α-linolenic acid. Fatty acids participate in the normal operation and metabolism of cholesterol in human body, inhibit the formation of arterial thrombosis, regulate human body functions and have other many functions such as prevention of cardiovascular disease, antibacterial and anti-inflammatory (55–57). The study showed that linoleic acid (C18:2) was the main fatty acid of buckwheat sprouts, the percentage of linoleic acid content boosted from 38.1 to 52.1% and total unsaturated fatty acids composition was more than 83% after 7 days of germination (28). Tartary buckwheat bran and flour were examined and quantified using GC–MS. The results indicated that a total of 8 fatty acids including myristic acid (C14:0), palmitic acid (C16:0), stearic acid (C18:0), oleic acid (C18:1), linoleic acid (C18:2n-6c), linolenic acid (C18:3n-3), arachidic acid (C20:0), and cis-11-eicosenoic acid (C20:1) were identified, in which C18:2n-6c, C18:1, and C16:0 were primary fatty acids of Tartary buckwheat (98). However, previously studied data showed the content of fatty acids in buckwheat seeds changed with germination days. For instance, the content of palmitic acid, stearic acid, eicosenoic acid and arachidonic acid increased gradually, while the linoleic acid content decreased (45). In terms of their functions, the changes of fatty acids during germination were also bidirectional. Twenty-five fatty acids were detected in sprouts of Tartary buckwheat, among which the amounts of palmitic acid, oleic acid, and linoleic acid were higher. During germination, sugar metabolism and fatty acid synthesis of Tartary buckwheat seeds would increase. Starch and other polysaccharides in the seeds were hydrolyzed to monosaccharides, which would be converted into energy and other biomolecules, including fatty acids. Therefore, increased sugar metabolism during germination could provide necessary carbon sources and energy for fatty acid synthesis. So, the content of total fatty acids (TFA) improved gradually with increasing germination time, while the content of stearic acid and oleic acid declined. The content of unsaturated fatty acids decreased first and then increased, and the value of UFA/TFA decreased. In general, the contents of TFA, UFA and some polyunsaturated fatty acids in edible Tartary buckwheat sprouts could be upgraded by germination, but the UFA/TFA value was reduced (83).

### 2.6. Tartary buckwheat minerals

Tartary buckwheat is rich in a variety of minerals beneficial to human health, especially potassium, sodium, copper, zinc, calcium and iron, which are much higher than other crops. Huang et al. found that the contents of Cu, Zn, Fe, K, and Mg elements were great fluctuation, those ranges were 5.74 ~ 36.01, 8.44 ~ 66.63, 21.8 ~ 3,990, 1737 ~ 5,831, and 729 ~ 3,104 mg/kg in the Tartary buckwheat seeds (water content about 13%) (8). The contents of Cu, Mn, Fe, Zn, K, Mg, and Ca in Tartary buckwheat flours varied from 0.64 to 2.81, 0.08 to 2.72, 1.75 to 17.21, 1.23 to 5.79, 280.6 to 648.7, 66.90 to 362.90, and 30.0 to 331 mg/100 g, respectively. K, Mg, and Ca were abundant in Tartary buckwheat varieties, of which had the highest content in B-121 (648.7 ± 11.54 mg/kg), IC-329200 (361.30 ± 2.02 mg/kg), and IC-274439 (331.0 ± 4.28 mg/kg), respectively. Due to the variety of Tartary buckwheat, the contents of mineral elements are very different (79).

During germination, the contents of various minerals in Tartary buckwheat will change in different degrees. In the process of plant growth, mineral elements play a significant role in the regulation of cellular permeability and osmotic balance, acting as enzyme cofactors (99) and forming transmembrane potentials (100). Furthermore, they produce significant effects on cell division and elongation, related enzyme activity, protein synthesis and respiration.

### 2.7. Tartary buckwheat germination as a tool to improve biological activity

Being a major source of rutin, buckwheat has a great deal of pharmacological functions, such as antioxidant and hypoglycemic effects (101). Germination can activate a variety of enzymes in plants, and many physiological and biochemical reactions occur during seed germination, resulting in a significant increase in the contents of functional active components such as flavonoid and phenol in the body, thereby enhancing the bud or seedling health effects.

#### 2.7.1. Antioxidant effect

Sim et al. indicated that sucrose and CaCl_2_ induction not only significantly enhanced the antioxidant capacity of buckwheat malt on HepG2 cells, but also protected fibroblasts from oxidative damage. CaCl_2_ could effectively maintain the accumulation of bioactive compounds in buckwheat sprouts, thus enhancing the sucrose-induced antioxidant capacity (102). The improvement of oxidative damage in HePG2 cells by buckwheat sprouts was mainly achieved by regulating the production of reactive oxygen species and malondialdehyde, and antioxidant enzyme activity (36).

Physical or chemical treatments can affect Tartary buckwheat seeds or seedlings to varying degrees. Besides, the ability and potential of germination and the activity of enzymes such as SOD and Peroxidase (POD) are improved to resist adversity, accelerate metabolism and photosynthesis, and promote the production of secondary metabolites (36, 97).

#### 2.7.2. Anti-inflammatory effect

When Tartary buckwheat was treated with laser light (He–Ne laser, 632 nm, 5 mW), the results indicated that laser enhanced the antioxidant capacity and anti-inflammatory activities by restraining the activities of cyclooxygenase-2 and lipoxygenase, particularly in sprouts (38). Another study suggested that according to the assessment of the production of proinflammatory mediators such as nitric oxide and cytokines including tumor necrosis factor-α, interleukin- (IL-) 6, and IL-12 in lipopolysaccharide- (LPS-) RAW 264.7 macrophages, the extracts of Tartary buckwheat sprout displayed higher inhibitory activity than that of common buckwheat sprout. Moreover, the production of LPS-induced cytokine in peritoneal macrophages decreased significantly under the effect of Tartary buckwheat sprout extracts (24). The above findings suggested that Tartary buckwheat sprout could be a potential source of anti-inflammatory agents. The experiments outcome confirmed that adipogenesis and inflammatory reaction during 3 T3-L1 cell differentiation could be inhibited by reducing glycerol-3-phosphate dehydrogenase (GPDH) activity and nitric oxide (NO: a plant signal) production, as well as by regulating the expression of genes related to fatty acid synthesis and inflammatory mediators. Consequently, Tartary buckwheat flavonoid can be applied to prevent inflammation caused by obesity (49).

#### 2.7.3. Hypoglycemic effect

The effects of purified total flavone extracted of Tartary buckwheat on insulin resistance and hepatic oxidative stress in mice was investigated. Tartary buckwheat could effectively prevent insulin resistance and oxidative stress induced by high fructose diet through some ways including lowering blood glucose and insulin concentration, improving insulin signaling molecules, restoring the altered antioxidant defense, and activating Nrf2 signaling pathway (103). D-chiralinositol (DCI) is a compound with insulin-like biological activity, whose free form can reduce the blood glucose and has hypoglycemic effect. Acting as a messenger during insulin signaling, directly promoting the combination of insulin and its receptor, thereby promoting insulin action and ultimately lowering blood glucose, DCI is a natural α-glucosidase inhibitor for diabetics (35). It has been reported that buckwheat DCI mainly exists in buckwheat phenol form, which can be converted into free DCI during germination (39). The research showed that the concentration of 0.05% NaHCO_3_ increased the DCI content in the sprouts of Tartary buckwheat by up to 9-fold (40), and metal ions (Al^3+^, Cu^2+^, Zn^2+^) also could increase α-glycosidase activity to release more free DCI, so as to increase the effect of reducing sugar in buckwheat (41).

## 3. Several new physical methods on germination of Tartary buckwheat seeds

The valid way to improve food nutritional value and flavor is to control germination (31). However, influenced by many intrinsic elements, seed germination is featured by a range of complicated physiological and biochemical processes (104). These factors consist of seed vigor, temperature, time, and some external stimuli like hormones, visible light, UV, US, MW, MF (30), ultra-high voltage, high voltage pulsed electric fields, ionizing radiation and pulsed MF. The new processing technologies are considered to be non-thermal processing. In contrast to thermal treatments, non-thermal techniques are not limited to altering the physical and chemical properties of grains, since through the induction of associated biochemical conversion, it is also possible to improve the nutrient and functional composition and reduce anti-nutrient factors (32).

US, MW, MF and other non-thermal technologies can stimulate cereal seeds germination and produce some specific effects, resulting in a series of physiological and biochemical changes, as well as attracting more and more researchers’ attention (Table 4).

**Table 4:** Facilitation effects of physical methods on seeds or sprouts.

Method	Facilitation effects	Researchers
Light	Increased the accumulation of total flavonoids and rutin	Lee et al. (105), Shin et al. (106), and Thwe et al. (107)
UV light (blue, white, green, yellow, red) blue light + UV	Increased the activities of PAL and CHI	Nam et al. (58) and Thwe et al. (107)
UV	Affected the biosynthesis of anthocyanins and procyanidins	Luo et al. (108) and Tatsuro et al. (109)
LED	Improved the nutrient quality in sprouts and microgreens, regulated flavonoids and carotenoids	Zhang et al. (110)
UV-B LED (blue and red light)	Regulated the accumulation of flavonoids	Luo et al. (111)
EMF SEF MF	Increased the activity of enzymes and the respiration rate, increased germination potential and germination rate, shortened germination time and promoted root growth	Dominguez et al. (112)
	Changed molecular structure and cell structure, increased seed coat permeability	Pittman et al. (113) and Zheng and Xu (114)
	Accelerated water and oxygen transport	Pittman et al. (113) and Zheng and Xu (114)
	Improved seed germination potential, germination rate, and seedling growth and quality	Pittman et al. (113) and Zheng and Xu (114)
	Improved seed germination vigor	Zhou et al. (96) and Payez et al. (115)
	Exhibited the synergistic effect of three enzymes (PAL, CHI and rutin-degrading enzymes) and the total flavonoids by 62.90 mg/g	Zhou et al. (96)
MW	Improved the germination; the accumulation of total flavonoids (57 mg/g), the content of sugar and soluble protein decreased, and scavenging activity of DPPH increased, free amino acid was up to 11 mg/g	Wang et al. (97)
	Altered biological macromolecular structure, affected the physiological and biochemical characteristics of plant cells, increased the penetration capacity of intracellular matter	Kouchebagh et al. (116)
	Loosened seed coat membranes, decreased their permittivity, and increased the electric conductivity	Hamada (117)
	Stimulated enzyme activity, promoted seed germination and accumulation of active substances	Jia et al. (118)
	Increased activity of antioxidant enzymes (SOD, POD, CAT, APX and GSH-Px)	Qiu et al. (119)
	Promoted the activities of nitrate reductase and glutamine synthetase and inhibited the activities of proteolytic enzymes and ribonuclease	Wu et al. (120)
	Increased alpha-amylase activity (up to 0.88 mg/g.min-1)	Chen et al. (121)
	Enhanced the seedlings ability and improved the resistance of seedlings to salt stress	Chen et al. (121)
	Affected the seeds vigor	Wang et al. (122)
	Increased the activities of PAL, CHI and FLS by 47.84, 53.04 and 28.02%	Peng et al. (10)
	Increased a 7.51-fold in reducing sugar, a 69.61% reduction in starch content, enhancements of PAL, CHI, FLS activities and flavonoid content by 5.15 g/100 g DW	Ma et al. (10)
US	Regulated the growth, physiological metabolism and killed pathogens of sprouts	Liu et al. (123)
	Promoted seed germination and plant growth	Mota et al. (124)
	Increased germination rate, sprout length, and content of GABA, daidzein and genistein in sprouts (soybean)	Yang et al. (125)
	Upregulated PAL, CHI, FLS genes, and increased the content of resveratrol *via* phenylpropanoid biosynthesis	Xie et al. (10)
	Caused seed coat fragmentation, promoted enzyme catalysis and accelerated seed germination	Yaldagard et al. (126)
lasers	Enhanced the nutritive values(49 targeted minerals, vitamins, pigments and antioxidants), boosted the antioxidant capacity and anti-inflammatory activities	Almuhayawi et al. (38)

### 3.1. Effect of light on germination of Tartary buckwheat seeds

As a prominent environmental factor, illumination effects in the accumulation of plant secondary metabolites. Light intensity and duration to a certain extent also have momentous impacts on plant growth (105). Compare with darkness, the germination, growth and flavonoid accumulation of Tartary buckwheat is more conducive in light ((105–107)). According to Lee’s experiment, the third day seedlings under dark conditions were treated with light-emitting diode (LED) lamps [red, blue and red + blue], results showed the rutin contents of Tartary buckwheat sprouts were documented 82% (*ca.* 29 mg/g DW) of the total phenolic compounds and which were around 6-fold higher than those of common buckwheat sprouts in both at 9 and 12 days after sowing (105). The common buckwheat was grown under different sources of light, it was found that the contents of the individual flavonoids in blue light-exposed were approximately 1.6 ~ 2.9-fold higher than that in the control group, meanwhile, the highest flavonoid content in blue light -exposed was observed for rutin (5.5 mg/g DW) (58). Besides, the treatment with appropriate intensity of single light (UV, blue, white, green, yellow and red)and combined light (blue and UV) significantly enhanced the activities of phenylalanine ammonia-lyase (PAL) and chalcone isomerase (CHI) in Tartary buckwheat seedlings (58, 107), as well as increased the accumulation of total flavonoids and rutin (105–107), and the activities of key enzymes involved in flavonoid synthesis were positively correlated with the accumulation of flavonoids and their antioxidant capacities (58). Being a plant artificial light source with innovation properties, LED has a broad application prospect in some aspects, such as improving the nutritional quality of plant sprouts and micro-green seedlings. Furthermore, it can light both as a supplemental source and a sole source. Some studies have initially determined that a whole string of structural genes involved in the biosynthesis of plant compounds (such as flavonoids and carotenoids) in buds and micro-green are regulated by LED light sources (110). Research has confirmed the biosynthesis of anthocyanins and procyanidins in Tartary buckwheat is affected by ultraviolet radiation (108, 109). A studied results showed that the distribution and content of flavonoids in cotyledons and hypocotyls were affected by different light sources, after the treatment of LED white light and LED blue light, the total flavonoids in Tartary buckwheat cotyledons at 3 days old sprouts were 2.12 and 2.73 times of those in the dark light group (*p* < 0.01) and the combination of UV-B, LED blue and red light was more appropriate to the regulation of flavonoids accumulation during the germination of Tartary buckwheat (111).

### 3.2. Effect of electromagnetic field on germination of Tartary buckwheat seeds

Treatment of plant seeds with EMF, SEF and MF can increase related enzymes activity and respiration rate at germination stage, reduce germination time, and promote root growth (112), upraised germination potential and germination rate of most crops. For example, seedling emergence rate produced significant increases of 69.2% with a dosage of 560 mT (112). The seed (Pot Marigold) with the treatment of MF from 5 to 15 min, the variety of the germination was of 86.33 to 20% (116). Electric and magnetic fields radiate plant seeds, which produce large amounts of heat after absorbing energy, causing a rapid rise in intracellular temperature and molecular structural changes such as partial bond breakage (113). This results in changes in cell structure and physiological and biochemical characteristics, increasing concentrations of free radicals in biofilms and permeability of seed coats. After the treatment with MF of 500 mT for 5 min, the germination energy and germination rate of wheat seeds were 93.6 and 95.8%, respectively, which increased by 10 and 7.4% compared with the control. Under the treatment of 200 mT for 30 min, the germination index and vigor index were 23.6 and 62.7, respectively, which increased by 58 and 35% compared with the control (114). Consequently, water and oxygen transport is speeded up (113, 114), improving seed germination potential and rate, promoting seedling growth and seedling quality, and improving metabolic function of crops, induction of plant resistance to increase the total yield of crops. Zhou et al. obtained that the effects of electric and magnetic fields on plant seeds vary with their properties and strengths, the biggest specific activity of PAL was attained at 0.3 T, whereas the specific activity of CHI reached the highest level at 0.2 T. Compared with the control, when the magnetic field intensity was 0.2, 0.3 and 0.4 T, the maximum increase of PAL enzyme activity was 2.4, 18.5 and 4.9%, respectively. However, the specific activity of CHI enzyme increased by 12.9 percent (0.2 T), 7.5 percent (0.3 T), and 7.4 percent (0.4 T), respectively (96). Accordingly, compared with the constant magnetic fields, EMF of the same intensity and time has more influence on seed germination (115). Wheat (115), soybean, corn (127) and buckwheat (96), are treated with high voltage electrostatic or MF, both of them can significantly improve seed germination vigor. The vary of MF intensities on the relationship between the specific activities of PAL, CHI and rutin-degrading enzymes and the total flavonoids accumulation was different during the germination of Tartary buckwheat seeds. The results exhibited that the synergistic effect of three enzymes was the best in 5-day-old seedling, and the total flavonoid accumulation was the most significant (62.90 mg/g) (96). So MF stimulation can induce the accumulation of flavonoid components in buckwheat seedlings.

### 3.3. Effect of MW on germination of Tartary buckwheat seeds

Being a kind of electromagnetic wave, MW is a rapid, convenient, economic and environmental non-thermal processing technology. Deng et al. found that MW (850 W) treatment processes decreased the starch, protein, fat, and ash contents. MW reduced the total flavonoids and essential amino acids and total amino by 19, 3.1 and 4.8%, respectively. Phytic acid and Trypsin inhibitor activity was lowered by 33.4 and 13%. The content of tannin and saponin lost 27.5 and 20.1%, respectively. But the protein digestibility and the ω-6/ω-3 ratio increased by 3.6 and by 4.9, respectively (128). Above outcome implied that low temperature MW treatment can alter biological macromolecular structure of plant seeds, and affect the physiological and biochemical characteristics of seed or seedling.

Under MW treatment, polar substances such as water, fat, carbohydrate and protein in plant cells can absorb electromagnetic energy, which may cause the change of the bond between molecules. Changes in molecular structure may result in changes in physical and chemical properties and morphological characteristics of plants (129). Therefore, MW treatment has the potential to improve the seed germination of various plants,. However, the exposure of MW for high intensity and duration time can inhibit seeds germination. The results showed that germination rate (600 W for 10 s, sowed 7 d) was 2 times that of the control, the germination rate for exposure of seeds to 800 W for 30 s was the lowest (*ca.*10%), a significant 87% reduction compared to the control group (97). Meanwhile, under condition of 600 W MW for 30 s, the content of the total flavonoids and free amino acid accelerated to 57 mg/g and 11 mg/g, respectively. The DPPH scavenging activity increased significantly, conversely, sugar and soluble protein levels were reduced (97). The results indicated that the germination rate of Tartary buckwheat seeds could be enhanced by suitable MW power treatment over a period of time, which probably resulted from the activation of activities of SOD and catalase (CAT) enzymes needed for growth and development by MW irradiation. At present, about the MW of Tartary buckwheat seeds grow less research on the effects of enzyme activity in the process (97). The reason of increasing the content of total flavonoids by MW treatment may be that it stimulates the activities of enzymes associated with phenylpropane metabolism, such as PAL, CHI and others. As previously mentioned, MW is also a kind of electromagnetic field (96).

In addition, it has been reported that the effect of MW treatment on seed germination is closely related to cracks on seed coat (130). The non-thermal effects of MW radiation are essentially biological. When interacting with the biological tissues, MW radiation will change the charge density on the cell membrane surface and the electric potential difference on both sides of the membrane, thus affecting the activity of ion channels, and ultimately promoting a range of plants physiological changes (131). Owing to plant seeds absorb MW energy, cells internal temperature rises swiftly, the pressure inside the cell surpasses the expansion capacity of cell wall, thus leading to cell rupture and free outflow of cell-active components. It represents an increased ability of soluble substances in cells to pierce through thin biofilm. Previous research has indicated that the alterations of Biomacromolecular structure and physiological and biochemical characteristics of plant cells are the result of the absorption of electromagnetic energy by polar molecules in plant cells (116). Exposed to MW doses between 840 and 1,260 W/g FW (fresh weight), the germination percentage of seeds was increased to 45%, the electrical conductivity of seed leachate was also increased by 28%, indicating seed coat membrane loosening. Observed seed coat by microscopic scanning, it was outcome that the treated seeds had disintegrated and facilitated water absorption. It also found that the least mobile water hydrated with macromolecules (bound water) appeared earlier in microwave-treated seeds than untreated seeds. When the seeds began to germinate, this enhanced hydration may trigger various metabolic activities (132). This kind of growth boost is probably due to irradiation breaking down sugars in the grains, providing nutrient substances for embryonic development, the study given that the content of protein and free amino acid content of 7-d-old wheat seedlings increased 38% (to 47 g/kg) and 6.13 times (to 14.05 g/kg) in the 75 min dose (117). Hence, proper MW processing can boost seed germination.

Studies on the effects of MW treatment on enzyme activity in cereal crops have shown that appropriate MW treatment can stimulate enzyme activity and promote seed germination and active substance accumulation in cereal crops (118). The study also showed that MW radiation treatment under appropriate conditions increased activity of antioxidant enzymes including SOD, POD, CAT, ascorbic peroxidase (APX) and GSH-Px (119). Similarly, MW pretreatment is effective in enhancing alpha-amylase activity (121). Although low-power MW processing facilitated the activities of nitrate reductase and glutamine synthetase in oat seed leaves, it also suppressed the activities of proteolytic enzymes and ribonuclease (120). Compared to the control, α-amylase activity in seedlings 24 h after MW pretreatment (10 s) has risen by 33.85% (up to 0.88 mg/g/min) (121). MW can effectively activate several enzymes associated with the metabolism in the process of germination. The suitable MW treatment can heighten the ability of wheat seedlings (variety Zhengzhou 9,023) to scavenge salt-stress-induced free radicals, thus improving the resistance of wheat seedlings to salt stress (121). The protection of MW processing is based on at least three mechanisms. First of all, it upgrades the activities of SOD, POD and CAT, which are regarded to be key enzymes in inhibiting the function of abiotic stress pathway. In the Second place, the concentrations of glutathione and ascorbic acid are improved, thereby eliminating free radicals and reducing oxidative stress. At length, it raises the concentration of NO, which reacts with free radicals such as O^- 2^ and H_2_O_2_.

The effects of MW treatment on the insecticidal efficacy and growth rate of wheat seeds were studied. The results showed that after exposure at 800 W MW for 20–25 s, the wheat seed germination rate significantly decreased by 25% and the seed vigor was inhibited. Thus, the authors hypothesize that the higher intensity MW (800 W) has deleterious effects to seeds (122).

Due to internal heating, MW treatment usually displays little potential in grain sterilization for sample damage caused by heating (133). Furthermore, having some shortcomings such as poor uniformity and heavy energy consumption, MW heating is not favorable to production on a large scale. However, the effects of low-power MW on physiological and biochemical indexes still need to be further studied. And studies have pointed out that MW radiation can dedicate to agriculture, industry and stock farming (120, 134).

The process of germination was accelerated at 300 W MW irradiation for 50 s. After germination, reducing sugar content increased by 7.51 times, starch content decreased by 69.61%, and amylase activity improved obviously. MW irradiation can enhance the activities of PAL, CHI and flavonol synthetase (FLS), and the total flavonoid content achieved the maximum value (5.15 g/100 g DW) after 300 W/50 s irradiation. Therefore, It is valid to improve the functional properties of edible Tartary buckwheat sprouts (83). Compared with CK, the specific activities of PAL, CHI and FLS in 5-day-old sprouts increased by 47.84, 53.04 and 28.02%, respectively, under the optimal treatment condition (MW 250 W, 90 s, 2.9 mmol/L L-Phe) (135).

### 3.4. Effect of ultrasound on germination of Tartary buckwheat seeds

As a kind of attractive and highly efficient non-thermal treatment technology, US can adjust the growth, physiological metabolism and kill pathogens of sprouts (123). In the applications of biotechnology, US is widely reported as an effective instrument for regulating and getting command of biological processes, including fermentation, enzymatic transformation (136), seed germination, and plant growth (124). But there is limited data on the physical and nutritional properties of Tartary buckwheat sprouts from US-treated seeds. In one study, soybean seeds were treated with US of varying powers (0 W to 300 W) and germinated in the dark for 5 d. The results indicated that US treatment increased germination rate (18.07%), sprout length (24.42%), GABA content (43.39%), daidzein (*ca.*79.62%) and genistein (*ca.*70.95%) contents of soybean sprouts (125). Upregulated three key genes namely the *arahy. Tifrunner. gnm1.ann1.DXZI51, arahy.Tifrunner.gnm1.ann1.VGN2GE*, and *arahy.Tifrunner. gnm1.ann1.Y23DM6* showed that resveratrol content could be increased by phenylpropanoid biosynthesis (137).

The “cavity effect” feature of US, MWs, and magnetization related to MF all influence plants growth and metabolism (138). Such physical treatments usually upgrade seed vitality and stimulate plant development simply by altering seed physiological process (138).

The proper and effective seed treatment is of great significance in enhancing germination rate and germination energy (GE), stimulating crop growth, as well as declining crop maturation period. Besides, the non-thermal treatment of MW, like EMF and MF, can improve seed GE and germination rate, enhance the quality and stress resistance of seedlings, and thus raise the yield and metabolic function of crops (139, 140).

Currently, although US, MW, and MF treatments are broadly used as complementary extraction and drying methods, few studies investigate their applications in grain germination (131). In addition, the US oscillations give rise to the rupture of seed coat, which boosts the hydration of dormant seeds. This treatment not only improves the water absorption of seeds but also effectively changes the structure of enzyme molecules by taking advantages of the cavitations and mass transport of enzymes, thus promoting the catalytic action of enzymes (aggrandized 5.74%) and accelerating seed germination (126). What’s more, this hydration has no adverse impact on seeds (126).

Current work indicates that US treatment can improve the edibility and nutritional quality of grain sprouts as an emerging way. These results for regulating the biosynthesis pathway of phenylpropanoid provide a certain reference, which can be used to regulate the nutritional and functional components of sprouted for human health benefits.

### 3.5. Effect of lasers on germination of Tartary buckwheat seeds

There are few studies on improving the nutritional value of buckwheat sprouts by laser. Almuhayawi et al. (38) treated common buckwheat and Tartary buckwheat with laser (He–Ne laser, 632 nm, 5 mW). Results showed that more that 35 of the 49 targeted minerals, vitamins, pigments and antioxidants were remarkably increased in common buckwheat and/or Tartary buckwheat sprouts. The laser light-treated common buckwheat and Tartary buckwheat sprouts, the total phenols content of sprouts magnified by 1.09 times (up to 12.19 mg/100 g FW) and 56.99% (up to 10.14 mg/100 g FW), respectively, meanwhile, the total flavonoids content of sprouts was augment by 1.78 times (up to 3.72 mg/100 g FW) and 183% (up to 5.46 mg/100 g FW), respectively. Besides, laser light improved the antioxidant capacity and anti-inflammatory activities by inhibiting cyclooxygenase-2 and lipoxygenase activities, especially in Tartary buckwheat sprouts. Hence, laser light has a great prospect in improving the nutritional and health-promoting values of buckwheat sprouts.

## 4. The mechanism of germination and enrichment of active components

### 4.1. The synthetic metabolic pathway of flavonoids

**Figure 1:**
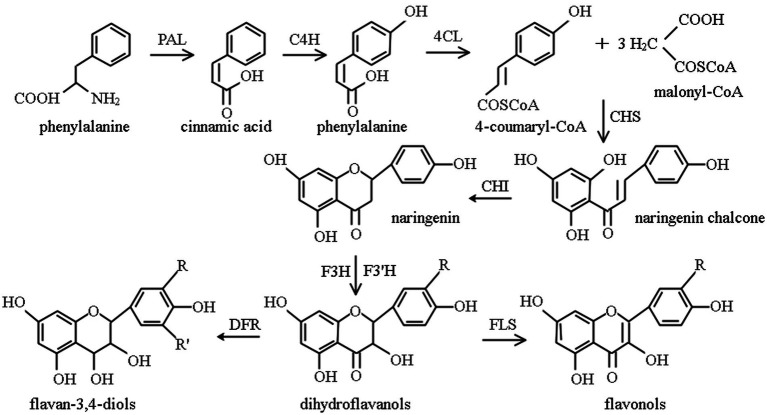
Phenylpropane metabolic pathway (141). PAL, phenylalanine ammonia-lyase; C4H, cinnamate 4-hydroxylase; 4CL, sweet bean acid coenzyme A ligase; CHS, chalcone synthase; CHI, chalcone isomerase; F3H, flavanone 3-hydroxylase; F3’H, flavonoid 3′-hydroxylase; DFR, dihydroflavonol reductase; FLS, flavonol synthase.

The synthesis and accumulation of flavonoid in plants depends on the activity of key enzymes in the flavonoid pathway, the phenylpropanoid pathway, as shown in Figure 1. Phenylpropanoid metabolism is an important pathway for the accumulation of secondary metabolites of flavonoids (81, 142), which is done under the catalysis of a series of enzymes (141). As three important enzymes in this pathway, the activities of PAL, CHI, and FLS act a significant role in the formation of secondary metabolites of flavonoids. Being the first enzyme involved in the flavonoid pathway, PAL is considered as the key rate-limiting enzyme in flavonoid biosynthesis. CHI controls the conversion efficiency of flavonoid precursors into flavanones, flavonoids, flavonols, anthocyanins, etc., their expression efficiency in plants directly affects the yield of total flavonoids. The highest content of flavonoids in plants is flavonol. FLS is a direct regulatory enzyme for flavonol synthesis, determining the content of total flavonoids in plants.

Chalcone is formed by the initial substrates 4-coumaryl-CoA and malonyl-CoA under the action of Chalcone Synthetase (CHS), and then CHI catalyzes chalcone to produce 4,5,7-trihydroxyflavanone, as a major product of metabolism, it re-enters other different metabolic pathways and forms different flavonoids (143). PAL and CHI are key enzymes in the biosynthesis of phenylalanine ammonia-lyase flavonoid, and their activity is tightly associated with flavonoids content in plants (144). PAL is a key enzyme and rate-limiting enzyme, connecting primary metabolism with phenylpropanoid metabolism, and catalyzing the first step of phenylpropanoid metabolism (145), its activity directly affects the cinnamic acid reserve and is closely related to the subsequent metabolism of flavonoids. Besides, it is the most studied enzyme in phenylpropanoid metabolic pathway (146). CHI is also a key enzyme and rate-limiting enzyme in phenylpropane metabolism that catalyzes the conversion of chalcone to highly bioactive flavonoids (145), and plays an important role in flavonoid metabolism (81). In flavonoid biosynthesis, being a key enzyme in flavonol bypass, FLS acts on the branching point of flavonoid and catalyzes the synthesis of flavonols, such as quercetin, using dihydroflavonols as substrates (147).

It has been shown that PAL and CHI are induced during seed germination by various external factors, such as light, mechanical damage, US, MW, and MF, etc. (148). In recent years, some new physical methods, such as ultrasound, laser, electric field, magnetic field and MW, are becoming more and more received for their advantages, including easy operation, short duration, low cost and environmental protection. In addition, having good biological effects, these methods show great advantages in improving the germination rate and growth vigor of Tartary buckwheat, enhancing the activity of enzymes related to growth metabolism and enriching bioactive substances. So, researchers at home and abroad have developed great enthusiasm for these emerging approaches (96, 97, 149). The application of these new methods has far-reaching significance to enrich bioactive components and increase yield of Tartary buckwheat during germination.

Light conditions have different effects on the activities of key enzymes for flavonoid enrichment. There are reports showed that UV-A (365 nm) UV-C (254 nm) and their combination treatments significantly increased the activities of key enzymes such as Phenylalanine ammonia-lyase, chalcone isomerase (CHI) and rutin degrading enzyme (RDEs) in Tartary buckwheat, and improved the content of total flavonoids (93, 94).

The appropriate intensity of electromagnetic field treatment can also improve the activities of related enzymes in plants. The changes of PAL and other three key enzyme activities during the germination of Tartary buckwheat seeds under different MF intensities was given (96), the specific activity of PAL was the highest at 0.3 t of MF, and that of CHI was the highest at 0.2 t of MF. The results showed that MF stimulation could induce the production of total flavonoids in Tartary buckwheat seeds by regulating the activities of key enzymes. Principal component analysis implied that changes in flavonoids, PAL, CHI, and FLS were highly correlative with antioxidant capacity and enzyme inhibitory activities.

### 4.2. Gene expression of key enzymes in flavonoid biosynthesis

It has been proved that the key enzyme activity and gene expression in the synthetic pathway are closely related to the environment in which plants live. Light quality, salt stress, and EMF all directly affect the expression of genes involved in plant information transduction and biosynthesis (111). Therefore, it is possible to regulate the activity of key enzymes and gene expression of flavonoid synthesis by different treatments of Tartary buckwheat, so as to achieve scientific and effective regulation of flavonoid synthesis (Table 5).

**Table 5:** Gene expression of key enzymes in flavonoid biosynthesis.

Method	Facilitation effect	Researchers
Light	Increased the activation of *FtPAL*, *FtCHS*, *FtF3H* and *FtANS*, promoted the activities of PAL, CHI and other related enzymes	Luo et al. (111)
Light and dark	Up-regulated the expression of *FtPAL* and *FtCHI* and the rutin content about 5 times higher	Thwe et al. (107)
Light (LED)	Increased the expression levels of genes (*FtDFR, FtANS* and *Ft4CL*) by 7.1-fold	Seo et al. (150)
Light (LED)	Increased the expression of *Ft4CL* to 61.6% (red light) and 220.8% (blue light), respectively Increased the expression of *Ft4CL* in cotyledons and hypocotyls by 185.0% Decreased the expression levels of *FtPAL* in hypocotyls by 9.3%	Luo et al. (111)
Light	Up-regulated the expression of *FtANR* and *FtLAR1 with light* (blue light, far red light and ultraviolet light) Up-regulated the expression of *FtLAR3* with red light Affected the expression of *FtANR, FtLAR1* and *FtLAR3 by* light quality	Jiang et al. (151)
	*MYB* regulated flavonoid biosynthesis	Schijlen et al. (152)
Jasmonic acid	Degraded *FtMYB13, FtMYB14* and *FtMYB15*, *FtSAD2* and *FtJAZ1*interacted with *FtMYB11, FtMYB13, FtMYB14* and *FtMYB15*to promote the repressor activity of *FtMYBs*	Zhang et al., (153) and Zhou et al. (154)
Germination	Accumulation of total flavonoids was positively correlated with *FtMYB3* and negatively correlated with *FtMYB2* expression	Zhao et al. (155)
MW (300 W/25–75 s)	Upregulated the transcription of flavonoid biosynthetic enzyme genes (*FtPAL*, *FtCHI*, and *FtFLS2*)	Ma et al. (156)
MW (300 W/50 s)	Produced 7, 5, and 5 differentially expressed proteins (DEPs) related to gene expression, and flavonoids metabolism	Wang et al. (157)
MW (250 W, 90 s, 2.9 mmol/ L L-Phe)	Increased the expression of *FtPAL*, *FtCHI* and *FtFLS1* by 39.84, 24.78, and 33.72%	Peng et al. (135)

The enhancement of flavonoid content under light processing may be affected by the activation of *FtPAL, FtCHS, FtF3H, and FtANS*, which promotes the activities of PAL, CHI and other related enzymes in phenylpropanoid metabolic pathway (111). Under light and dark conditions, the expression level of flavonoid synthesis pathway genes could be increased, and higher levels of phenolic compounds, rutin and two anthocyanins were detected, the expression of *FtPAL* and *FtCHI*is significantly up-regulated, and the rutin content is about 5 times higher (107). A report also demonstrated that LED treatment could increase the expression levels of regulatory genes such as *FtDFR, FtANS* and *Ft4CL* by 7.1-fold, the content of phenolic compounds in Tartary buckwheat also increased with the use of LED lights (150). Compared with dark treatment, different light quality treatments alone and in combination could significantly regulate the gene expression of key enzymes in flavonoid metabolism pathway in Tartary buckwheat. The other studied results showed that the sensitivity of different tissues of Tartary buckwheat to different wavelengths of light and the expression of *Ft4CL* in cotyledons treated with different red light were significantly increased to 61.6% (*p* < 0.01) (111). However, the expression level of *Ft4CL* in the cotyledons treated with LED blue light was significantly increased by 220.8% (*p* < 0.01). The expression levels of *Ft4CL* in cotyledons and hypocotyls were significantly increased by 185.0% (*p* < 0.05), and the expression levels of *FtPAL* in hypocotyls were significantly decreased by 9.3% (*p* < 0.05). Another studied that the effects of light quality on ANR, a key synthetic gene in procyanidin metabolism pathway, the expression of *FtANR* and *FtLAR1* was up-regulated after the treatment of blue light, far red light and ultraviolet light, and the expression of *FtLAR3* was up-regulated after the treatment of red light (151). This result showed that different light quality could affect the expression of *FtANR, FtLAR1* and *FtLAR3* in different degrees.

*The myeloblastosis oncogene (MYB)* transcription factor is notable in flavonoid biosynthesis in buckwheat, which is involved in multiple metabolic pathways of flavonoid biosynthesis and can coordinate with multiple genes to regulate flavonoid biosynthesis (152). Studies have confirmed that *FtSAD2* (sensitive to abscisic acid and drought) and *FtJAZ1* (inhibitor of JA signaling) can interact synergistically with *MYB*, thus influencing the accumulation of flavones in Tartary buckwheat (154). The other study confirmed that *Fagopyrum tataricum MYB* transcription factors *FtMYB13, FtMYB14* and *FtMYB15* were degraded by jasmonic acid at protein level, which could directly inhibit the expression of *FtPAL* gene and reduce the accumulation of flavonoids. In addition, *FtSAD2* and *FtJAZ1*, which interact with *FtMYB11, FtMYB13, FtMYB14*, and *FtMYB15*, could significantly promote the repressor activity of *FtMYBs* (protein–protein interaction), thereby affecting flavonoid synthesis (153, 154).

In the cotyledons, the synthetic accumulation of total flavonoids was positively correlated with *FtMYB3* and negatively correlated with *FtMYB2* expression (155). However, the correlation between PAL, CHI and FLS and *FtMYB1, FtMYB2* and *FtMYB3* was more complicated, and the regularity between the transcription of key enzyme genes and the expression of transcription factors was not strong. It is inferred that a key enzyme gene of flavonoid synthesis may only be regulated by a specific transcription factor, which precisely opens one or more branches of flavonoid synthesis, the change of total flavonoid content can not reflect this characteristic.

It was found that these key enzyme-encoding contained a great quantity of light-response elements and hormone-response elements through promoter analysis. Furthermore, *MYB* transcription factors regulated the transcription of the target gene by direct combination with the promoter region of the target gene, thus regulating the synthesis of flavonoids. Yao et al. (158) identified 48 key enzyme-encoding genes involved in flavonoid metabolic pathways, and clarified the structure, evolution and tissue-specific expression patterns of these genes. Therefore, it also explained why light and hormones can induce the expression of flavonoid synthesis-related enzymes in Tartary buckwheat and improve the flavonoid content.

At present, in Tartary buckwheat germination, the MW technology have been applied, the less research of regulatory mechanism of gene expression of enzymes related to flavonoid synthesis is done. The study confirmed that in Tartary buckwheat sprouts, the transcription of various flavonoid biosynthetic enzyme gene containing *FtPAL*, *FtCHI*, and *FtFLS2* was obviously raised by MW irradiation (300 W/25 ~ 75 s) during cultivation. After 300 W/75 s MW irradiation, the maximum rutin content (25064.19 mg/kg DW) was observed in 5-day-old sprouts, while the maximum chlorogenic acid (497.95 mg/kg DW) and vitexin (345.75 mg/kg DW) were observed after 300 W/50 s MW irradiation (156). The effect mechanism of MW treatment on the germination and flavonoids enrichment of Tartary buckwheat was revealed (157). Besides, label-free quantitative proteomic analysis showed that after the treatment of 300 W/50 s MW, Tartary buckwheat had 7, 5, and 5 differentially expressed proteins (DEPs) for 3 ~ 7 days germination compared with the control group. These DEPs were primarily relevant to energy production and conversion, gene expression, and flavonoids metabolism. Based on KEGG (Kyoto encyclopedia of genes and genomes) analysis, the DEPs were mainly concentrated in photosynthesis, RNA polymerase, flavonoid biosynthesis, phenylalanine metabolism, and phenylpropanoid biosynthesis metabolic pathways. Furthermore, the upregulation of phenylalanine ammonia-lyase and flavonol synthase protein enzymes promoted germination and flavonoids accumulation in Tartary buckwheat. Another study showed that compared with seeds, chlorogenic acid and rutin content in 7-day-old sprouts increased by 13420.63 and 225.12%, respectively (135). And under the best treatment condition (MW 250 W, 90 s, 2.9 mmol/L L-Phe), the specific activities of PAL, CHI and FLS in 5-day-old sprouts increased by 47.84, 53.04 and 28.02% compared with CK, respectively, and the expression of *FtPAL*, *FtCHI* and *FtFlS1* increased by 39.84, 24.78 and 33.72% compared with CK, respectively. Hence, MW irradiation is a valid technique to enhance the functional attributes of edible Tartary buckwheat sprouts, but its power condition and exposure time need to be better optimized and determined whether it can promote seed germination.

US and MF treatments are widely used to cereal germination (33), but less used for the key enzymes enrichment of flavonoids in Tartary buckwheat. The regulatory mechanism of these treatments on gene expression of related enzymes is not clear, and further research is needed.

## 5. Summary

After buckwheat seeds germinated into sprouts, the nutritional value of buckwheat seeds was greatly increased, the trypsin inhibitor activity was low or even disappeared, the proportion of protein and amino acids was more balanced, the nutritional value of fatty acids was increased, and the activity of rutin-degrading enzyme disappeared, especially the content of flavonoids increased obviously. Under adversity, plants produce a large number of flavonoids, GABA and other secondary metabolites to repair the damage caused by adversity. In recent years, the research on the enrichment of flavonoids in buckwheat is limited to the effect on its content, but the research on its biosynthesis and regulation genes is lacking. Therefore, the key regulatory factors in flavonoid synthesis need to be further studied and explored. At the same time, there is still much room for further research on the physiological functions and mechanisms of flavonoid in plants. In the future, researchers can study the mechanism of buckwheat flavonoids enrichment, such as the activities of related endogenous enzymes, the expression of key enzyme genes and key regulatory factors, so as to accumulate specific secondary metabolites, and thus more effectively play unique health care value of buckwheat flavonoids. Through animal experiments, the health effects of buckwheat sprouts can be further clarified, which provides a theoretical basis for the development of deep-processed sprout vegetables with various health functions. The research on these problems has far-reaching significance for promoting the healthy and rapid development of buckwheat industry in our country.

## Author contributions

YD and NW: conceptualization. WP, NW, JW, YD, and SW: validation. YD: writing – original draft preparation. SW: writing – review and editing, supervision, project administration, and funding acquisition. WP: visualization. All authors have read and agreed to the published version of the manuscript.

## Funding

This research was funded by National Natural Science Foundation of China, grant number 31772025 and Anhui Natural Science Foundation, grant number 1808085MC93.

## Conflict of Interest

The authors declare that the research was conducted in the absence of any commercial or financial relationships that could be construed as a potential conflict of interest.

## Publisher’s note

All claims expressed in this article are solely those of the authors and do not necessarily represent those of their affiliated organizations, or those of the publisher, the editors and the reviewers. Any product that may be evaluated in this article, or claim that may be made by its manufacturer, is not guaranteed or endorsed by the publisher.
